# Sociodemographic, Behavioural, and Health Factors Associated with Sedentary Behaviour in Community-Dwelling Older Adults: A Nationwide Cross-Sectional Study

**DOI:** 10.3390/jcm12155005

**Published:** 2023-07-29

**Authors:** Dong Kee Jang, Mina Park, Yeo Hyung Kim

**Affiliations:** 1 Department of Internal Medicine, Seoul Metropolitan Government Boramae Medical Center, Seoul National University College of Medicine, Seoul 07061, Republic of Korea; mapmap05@snu.ac.kr; 2 Department of Rehabilitation Medicine, College of Medicine, The Catholic University of Korea, Seoul 06591, Republic of Korea; mmaabb@catholic.ac.kr

**Keywords:** aged, comorbidity, exercise, obesity, sedentary behaviour, sociodemographic factors

## Abstract

Few studies have focused on factors associated with sedentary behaviour among older Asian adults. This study aimed to identify factors independently associated with prolonged sedentary times in Korean older adults. We included 8273 community-dwelling older adults aged ≥65 years who participated in the Korean National Health and Nutrition Examination Survey. Self-reported sedentary times were assessed via the Global Physical Activity Questionnaire, and sedentary times of ≥420 min/day were considered ‘long’. Complex-sample multivariable-adjusted logistic regression analyses were conducted to investigate the factors associated with long sedentary times. Among the participants, 4610 (55.72%) had long sedentary times that were associated with advanced age (odds ratio [OR], 2.49; 95% confidence interval [CI], 2.05–3.01), female sex (OR, 1.32; 95% CI, 1.11–1.57), unemployment (OR, 1.23; 95% CI, 1.09–1.38), living alone (OR, 1.24; 95% CI, 1.08–1.43), urban residence (OR, 1.35; 95% CI, 1.14–1.61), and insufficient aerobic exercise (OR, 1.80; 95% CI, 1.60–2.02). Among health factors, obesity (OR, 1.27; 95% CI, 1.12–1.45), diabetes (OR, 1.17; 95% CI, 1.04–1.32), cardiovascular diseases (OR, 1.30; 95% CI, 1.11–1.52), and arthritis (OR, 1.26; 95% CI, 1.11–1.43) had positive associations with long sedentary times. A tailored approach that considered various sociodemographic, behavioural, and health factors is needed to reduce sedentary behaviour in this population.

## 1. Introduction

Sedentary behaviour refers to any behaviour performed while awake that is characterised by an energy expenditure of ≤1.5 metabolic equivalents (METs) while in a sitting, reclining, or lying posture [1]. Sedentary behaviour has become increasingly prevalent in society, and older adults are particularly susceptible to sedentary lifestyles [2]. Age-related physical changes, such as decreased muscle mass, as well as chronic health conditions, such as arthritis and cardiovascular disease, can contribute to increased sedentary times in older adults. The consequences of greater sedentary times for the older population include a higher risk of all-cause mortality, detrimental health outcomes, and poor mental well-being [3,4,5]. Increased sedentary behaviour can also contribute to muscle weakness, reduced bone density, and increased frailty in older adults, with these consequences occurring independent of physical activity [6,7].

A previous study described an ecological model of the determinants of sedentary behaviour in adults to provide multiple levels of influence, including individual, social, organisational/community, environmental, and policy [8]. Furthermore, the systems involved in the sedentary behaviour framework were developed based on international transdisciplinary consensus [9]. This framework consisted of six clusters of determinants: physical health and well-being, social and cultural context, built and natural environment, psychology and behaviour, politics and economics, and institutional and home settings. However, an ecological model of sedentary behaviour specific to the older population has not been suggested. Furthermore, current evidence regarding the factors associated with sedentary behaviours is too weak to develop a robust ecologic model.

A few studies indicated that unmarried status, lower education level, obesity, and a history of smoking were associated with increased sedentary times in old age [10,11]. A systematic review reported consistent positive associations between sedentary behaviour and age and consistent negative associations between sedentary behaviour and good retirement, obesity, and health status [12]. Lower educational level, obesity, and living in rural areas were also suggested as markers of high sedentary times in adults [13,14]. Additionally, sedentary behaviour is known to be associated with chronic diseases, such as cardiovascular disease, diabetes, certain types of cancer, and mental health issues [3,15,16]. Since most of these studies have assessed the markers of long television viewing times, it would be valuable to identify the markers of long total sedentary times. The individuals with long total sedentary times would be those who have high risk of poor health outcomes, and these individuals can be the primary target population for interventions [8].

To identify sedentary older individuals and encourage physical activity, understanding the complex factors associated with sedentary behaviour is imperative. These factors encompass multiple levels of influence, including individual, social, organisational/community, environmental, and policies. As noted in earlier studies, especially among older individuals, evidence of the determinants of sedentary behaviour is lacking, and most previous studies primarily focused on several individual factors and television viewing times [8,12]. Moreover, most factors known to be associated with sedentary behaviour in older populations to date are based on evidence obtained from studies conducted in Western countries. Therefore, the current study aimed to determine the sociodemographic, behavioural, and health factors associated with long total sedentary times in the Korean older population (aged ≥65 years) using a nationally representative database and relevant statistics. Our findings can contribute to the development of more robust ecologic models of the determinants of sedentary behaviour in the older population.

## 2. Materials and Methods

### 2.1. Study Participants

The Korea Centers for Disease Control and Prevention (KCDC) annually conducts the Korean National Health and Nutrition Examination Survey (KNHANES) to assess the health status, health behaviours, and food and nutrition intake of Korean individuals who reside in the community. The KNHANES consists of health and nutrition surveys and physical examinations. Health surveys and physical examinations are conducted in mobile examination centres, whereas nutrition surveys are conducted via household visits. The KCDC provides de-identified KNHANES data to the public on its website (https://knhanes.kdca.go.kr/, accessed on 10 January 2023). All participants provided written informed consent before participating in the KNHANES.

The current study utilised data derived from KNHANES VI, VII, and VIII, which were collected between 2014 and 2019. Among 47,309 participants (21,566 men and 25,743 women), 9825 participants were aged 65 or above (4240 men and 5585 women). After excluding participants who were missing data related to sedentary behaviour, the present study included 8273 participants (3676 men and 4597 women). This study, which utilised anonymised data available to the public, was granted exemption from ethical approval by the Institutional Review Board of Uijeongbu St. Mary’s Hospital.

### 2.2. Sedentary Behaviour and Physical Activity

Sedentary times and aerobic exercise were evaluated using a validated Korean version of the World Health Organization (WHO) Global Physical Activity Questionnaire (GPAQ) [17,18,19,20]. Sedentary times was assessed using question 16 of the GPAG: ‘How much time do you usually spend sitting or reclining on a typical day?’ Sedentary behaviour included sitting or reclining while at work and home; traveling to and from places, sitting with friends; travelling in a car, bus, or train; reading; playing card; or watching television. Time spent sleeping was not included in the sedentary behaviour survey. ‘Long’ sedentary times were defined as sedentary times of ≥420 min/day; otherwise, they were defined as ‘short’ sedentary times [21].

The GPAQ includes the time spent taking part in aerobic activities that require an increase in breathing or heart rate during a typical week. Following the WHO global recommendations on physical activity for health, the aerobic exercise levels of older adults were classified as sufficient if the participants met any of the following three criteria: (1) performing ≥75 min/week of vigorous-intensity aerobic activity; (2) performing ≥150 min/week of moderate-intensity aerobic activity; and (3) performing an equivalent combination of moderate- and vigorous-intensity activity. An insufficient level of activity was categorised as meeting none of the above three criteria [22].

Resistance exercise was assessed using the question: ‘How many days in the past week did you engage in resistance exercises such as push-ups, sit-ups, weightlifting, gymnastics, and pull-ups?’ According to the WHO global recommendations on physical activity for health, participants who performed resistance exercises on at least 2 days per week were categorised as completing sufficient resistance exercise [22]. Participants who performed resistance exercises on fewer than 2 days per week were classified as completing insufficient resistance exercise.

### 2.3. Variables

Age was classified into the following categories with 5-year intervals: 65–69, 70–74, 75–79, and ≥80 years. Excessive alcohol consumption was defined as over 20 g/day for men and 10 g/day for women. The following demographic information was obtained from the participants: smoking habits (never smoked, past smoker, or current smoker), educational level (>9 years or ≤9 years), occupational status (employed or unemployed), marital status (married or unmarried), household composition (living with others or living alone), household income (low-, lower-middle-, upper-middle-, or high-income household), and place of residence (rural or urban). Following the WHO guidelines for adult Asians, an individual’s weight status was classified as underweight (body mass index [BMI] < 18.5 kg/m^2^), normal weight (18.5 kg/m^2^ ≤ BMI < 23 kg/m^2^), overweight (23.0 kg/m^2^ ≤ BMI < 25 kg/m^2^), or obese (BMI ≥ 25 kg/m^2^).

Systolic and diastolic blood pressure, HbA1c level, and fasting blood glucose level were assessed using objective methods by trained nurses. The blood pressure was measured using a mercury sphygmomanometer (Baumanometer^®^, Wall Unit 33(0850), W.A. Baum, Copiague, NY, USA). Venous blood samples from participants were analysed within 24 h in the central laboratory. Participants were considered to have hypertension if their systolic blood pressure was ≥140 mmHg, if their diastolic blood pressure was ≥90 mmHg, or if they were taking antihypertensive medication. Individuals with diabetes were defined as having a haemoglobin A1c (HbA1c) level of ≥6.5%, having a fasting blood glucose level of ≥126 mg/dL, having received a diagnosis of diabetes from a doctor, or taking oral hypoglycaemic agents or insulin. Participants who had been diagnosed with stroke, myocardial infarction, or angina by a doctor were considered to have cardiovascular diseases. Patients who had previously been diagnosed with cancer, arthritis (osteoarthritis or rheumatoid arthritis), or depression by a doctor were considered to have cancer, arthritis, or depression, respectively. The number of comorbidities was determined by counting the number of diseases, including hypertension, diabetes, cardiovascular disease, cancer, arthritis, and depression.

### 2.4. Statistical Analysis

As the KNHANES data were collected through a sample survey, rather than through a complete enumeration survey, a two-stage stratified cluster sampling design was used to interpret the study’s results to fit the entire Korean population. We used a complex sampling design and incorporated sampling weights in all analyses to account for inclusion errors, uneven sampling rates, and non-response errors in the KNHANES. The characteristics of the participants according to sedentary behaviour status were compared using a complex-sample chi-square test. Multivariable-adjusted complex-sample logistic regression analyses were used to evaluate the associations between sociodemographic, behavioural, and health factors and sedentary behaviour. The prevalence of sedentary participants according to the presence of each comorbid disease was analysed using a complex-sample chi-square test. Considering the emerging emphasis on the role of multimorbidity in influencing health outcomes and potential association between multimorbidity and sedentary behaviours [23,24], additional analyses of the associations between the number of comorbid diseases and sedentary behaviour were performed. The prevalence of sedentary individuals based on the number of comorbidities was compared via a complex-sample chi-square test. The association between the number of comorbidities and long sedentary times was evaluated via multivariable-adjusted complex-sample logistic regression analyses. SPSS version 24 (IBM/SPSS Inc., Armonk, NY, USA) was used to perform statistical calculations. Statistical significance was considered as *p* < 0.05.

## 3. Results

### 3.1. Participant Characteristics

Among the 8273 older adults who resided in the community, 4610 (55.72%) individuals had long sedentary times, i.e., daily sedentary times of ≥420 min. The characteristics of the participating older adults are shown in Table 1. Participants with long sedentary times tended to be older, be female, be unemployed, live alone, have low household incomes, and live in urban areas to a greater extent than participants with short sedentary times. The prevalence of participants with insufficient aerobic exercise, insufficient resistance exercise, obesity, hypertension, diabetes, cardiovascular diseases, arthritis, and depression was significantly higher in older adults with long sedentary times than in older adults with short sedentary times. The education level, marital status, alcohol consumption, smoking habits, and prevalence of cancer were not significantly different between the two groups of participants.

### 3.2. Sociodemographic Factors Associated with Sedentary Behaviour

Table 2 shows the association between sociodemographic factors and long sedentary times.

Before adjusting for potential confounders, older age, female sex, being unemployed, living alone, having a middle-level household income, and living in an urban area were significantly associated with long sedentary times. After adjusting for other sociodemographic, behavioural, and health factors, the association between the following factors and long sedentary times remained significant: age ≥ 80 years (odds ratio [OR], 2.49; 95% confidence interval [CI], 2.05–3.01), age of 75–79 years (OR, 1.48; 95% CI, 1.26–1.73), age of 70–74 years (OR, 1.15; 95% CI, 1.01–1.31), female sex (OR, 1.32; 95% CI, 1.11–1.57), unemployed status (OR, 1.23; 95% CI, 1.09–1.38), living alone (OR, 1.24; 95% CI, 1.08–1.43), and urban residence (OR, 1.35; 95% CI, 1.14–1.61). Meanwhile, a higher level of education became a significant marker of sedentary behaviour after adjusting for confounders. The nature of the association between household income and sedentary behaviour was reversed after multi-variable adjustment. The marital status of older individuals was not associated with long sedentary times.

### 3.3. Behavioural and Health Factors Associated with Sedentary Behaviour

Among the behavioural factors, insufficient aerobic exercise was associated with long sedentary times (Table 3). This positive association remained significant after adjusting for other behavioural, sociodemographic, and health factors (OR, 1.80; 95% CI, 1.60–2.02). The positive association between insufficient resistance exercise and sedentary behaviour became insignificant after adjusting for confounders, whereas the association between smoking and sedentary behaviour became significant after adjusting for multiple variables. Excessive alcohol consumption was not associated with long sedentary times.

Table 3 also shows the association between health factors and prolonged sedentary times. Obesity (OR, 1.27; 95% CI, 1.12–1.45) and being overweight (OR, 1.17; 95% CI, 1.01–1.34) were independently associated with long sedentary times. Among the major comorbid diseases, diabetes (OR, 1.17; 95% CI, 1.04–1.32), cardiovascular disease (OR, 1.30; 95% CI, 1.11–1.52), and arthritis (OR, 1.26; 95% CI, 1.11–1.43) were significantly associated with long sedentary times. However, the associations between hypertension and depression and sedentary behaviour became insignificant after adjusting for confounders.

**Figure 1:**
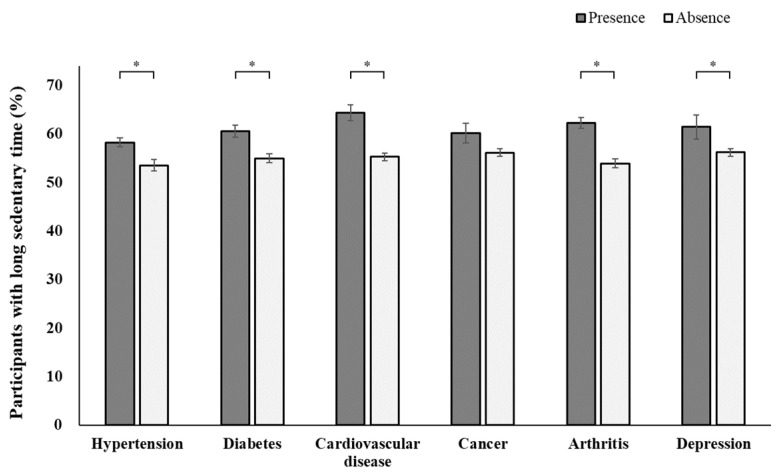
The prevalence of long sedentary times based on the presence of each comorbidity. * *p* < 0.05.

**Figure 2:**
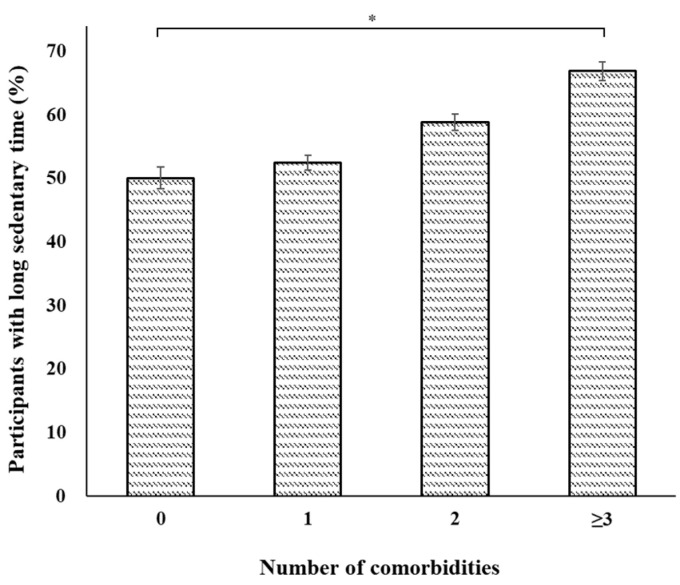
The weighted prevalence of long sedentary times based on the number of comorbidities. * *p* < 0.05.

**Figure 3:**
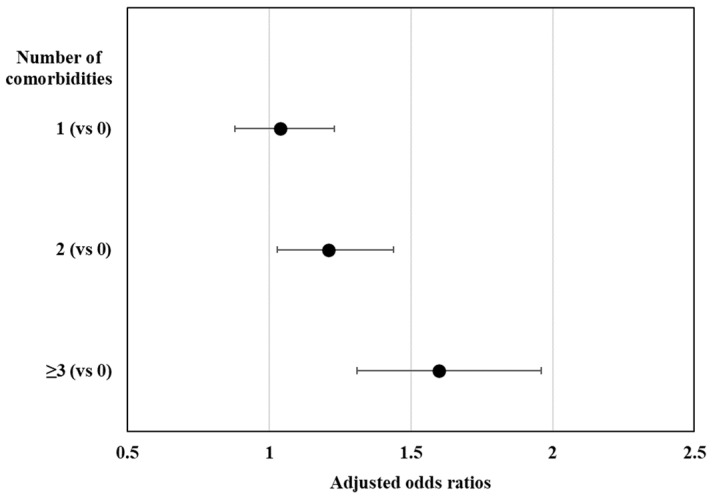
Association between the number of comorbidities and long sedentary times. Values are odds ratios adjusted for age, sex, education, occupation, marital status, household composition, household income, residence, alcohol, smoking, aerobic exercise, resistance exercise, and obesity.

Figure 1 shows the prevalence of participants with long sedentary times based on the presence of major comorbidities. The prevalence of long sedentary times was significantly higher among participants with hypertension (58.26% vs. 53.52%; *p* < 0.001), diabetes (60.52% vs. 54.99%; *p* < 0.001), cardiovascular disease (64.37% vs. 55.30%; *p* < 0.001), arthritis (62.23% vs. 53.90%; *p* < 0.001), and depression (61.44% vs. 56.20%; *p* = 0.039) than in individuals without those respective diseases. The prevalence of long sedentary times significantly increased in individuals with a higher number of comorbidities (Figure 2; *p* < 0.001). As shown in Figure 3, after adjusting for multiple confounders, higher numbers of comorbidities were significantly associated with long sedentary times: values for individuals with ≥3 comorbidities (OR, 1.60; 95% CI, 1.31–1.96), 2 comorbidities (OR, 1.21; 95% CI, 1.03–1.44), and 1 comorbidity (OR, 1.04; 95% CI, 0.88–1.23) compared to those of individuals with no comorbidity.

## 4. Discussion

The current study revealed that older age, female sex, unemployed status, living alone, and urban residence were independently associated with long sedentary times in older adults. In addition, an insufficient level of aerobic exercise and the presence of obesity, diabetes, cardiovascular disease, and arthritis were positively associated with sedentary behaviour in older individuals after adjusting for multiple potential confounders. As a large-scale nationwide study, this study provides reliable evidence regarding the factors associated with sedentary behaviour in Korean older adults. Furthermore, the determined sociodemographic, behavioural, and health factors encompass physical health and well-being, social and cultural context, built and natural environment, psychology and behaviour, and institutional and home setting clusters of systems in the sedentary behaviour framework. Identifying the factors associated with sedentary behaviour in older adults is essential to designing tailored interventions, preventing health risks, and promoting active lifestyles. By determining these factors, individuals, communities, clinicians, and policymakers can collaborate to support and promote healthier aging in older adults.

The results of this study, which indicate that sedentary times increase with age, are consistent with those of most previous studies [12,25,26,27]. Conversely, a study conducted in the United Kingdom reported no association between age and sedentary behaviour. However, this result could be confounded by the characteristics of the study participants, who were drawn from an exercise promotion trial [11]. Our study showed that as age increased, the effect estimates of long sedentary times increased, and the OR was 2.82 in the age group of ≥80 years compared to the 65–69-year (reference) age group. Therefore, healthcare professionals who specialize in the geriatric population should consider the likelihood of long sedentary times in the oldest of the older adult population [28]. To date, the research findings regarding the association between sex and sedentary behaviour in the older population have been inconsistent [12]. One potential explanation for women showing higher odds of sedentary behaviours in this study than in other studies could be related to differences in culture, ethnicity, and race. Another possible factor could be the higher proportion of women in the oldest age group; in Korea, women generally have longer lifespans than men [29].

The result of this study that indicates that unemployment has an independent association with long sedentary times is consistent with the findings of previous studies [12]. However, further research is needed to explore the effect of specific classifications of unemployment, including retirement, in older populations [30]. Although there have been numerous studies on individual factors associated with sedentary behaviour, there is a lack of research into the associations with interpersonal factors. One study conducted in Japan reported a 26% increase in television viewing times among individuals living alone compared to those who live in shared accommodation, which is consistent with the findings of our study [31]. Limited studies have been conducted regarding the association between environmental factors (including residential area) and sedentary behaviour, and research findings are not consistent between studies. Our study revealed that urban residency has an independent association with long sedentary time, which is consistent with the results of a study conducted in Belgium [32], but not with the results of a study conducted in Japan [31]. This inconsistency may be attributable to differences in the definition of sedentary time, such as whether studies considered total sedentary times or television viewing times.

The current study revealed that among the behavioural factors, insufficient aerobic exercise showed an independent association with longer sedentary times. Our findings are consistent with those of earlier studies that found an inverse relationship between moderate-to-vigorous physical activity and recreational walking or cycling and sedentary behaviour in older individuals [31,32]. In addition, the current study showed that obesity, diabetes, and cardiovascular disease were independently associated with long sedentary times, a result that is similar to the findings of previous studies [10,11,33,34]. Although a cause-and-effect relationship cannot be determined, the independent associations between insufficient aerobic exercise, obesity, and diabetes and sedentary behaviour suggest the possibility of a shared pathophysiology among these factors. All of these factors are connected to impaired insulin sensitivity, which is a prominent characteristic of type 2 diabetes. Aerobic exercise, through various molecular pathways, such as the upregulation of insulin transporters in insulin-dependent cells’ cellular membranes, has the potential to enhance insulin sensitivity [35]. Inflammation acts as a potential link between obesity and impaired insulin sensitivity based on the release of inflammatory mediators by adipose tissue [36]. Sedentariness also decreases insulin sensitivity in muscle, which results in hyperinsulinemia occurring to maintain normal glucose disposal [37].

In our study, an independent association between resistance exercise and sedentary behaviour was not observed. To the best of our knowledge, there is scarce literature regarding the relationship between resistance exercise and sedentary behaviour, which makes it difficult to compare our results to those of previous studies. However, in the current study, the significant association between resistance exercise and long sedentary times disappears when various confounding variables are adjusted, which suggests that the association between resistance exercise and sedentary behaviour is influenced by other factors. Meanwhile, we only assessed the number of days on which the participants engaged in resistance exercise using a questionnaire. Since we were unable to capture the intensity or duration of the resistance exercise, the impact of resistance exercise on sedentary behaviour may not be fully evaluated.

Physical inactivity and sedentary behaviour are closely related, though they are distinct concepts in terms of human movement and health. While they both involve low levels of physical activity, they have different implications and effects on overall well-being. While physical inactivity specifically refers to insufficient engagement in recommended physical activity levels, sedentary behaviour encompasses a broader range of activities that involve prolonged sitting or reclining with minimal physical effort [38]. Researchers have employed the bedrest model to separately explore the impact of sedentary behaviour and physical activity. Findings from bedrest studies indicate that while exercise training can help to mitigate the decline in muscle mass and function, extensive amounts of exercise alone cannot completely reverse the unfavourable metabolic changes induced via physical inactivity [39,40,41].

Our study indicated that arthritis was independently associated with sedentary behaviour after adjusting for age and other multiple confounders. Arthritis, especially that involving the lower extremity joints, generally results in pain and difficulty moving; therefore, patients with arthritis are commonly perceived as being more sedentary. Although previous studies have reported that sedentary times are long in patients with osteoarthritis or rheumatoid arthritis [42,43], few studies have compared the sedentary behaviour of this population to matched control groups. In a cross-sectional study of older women, no significant difference was found in the mean total sedentary times between women with and without knee osteoarthritis [44]. Although this finding is not consistent with the findings of our study, that study did not adjust for confounding factors and simply compared the mean sedentary time. Considering that pain can be exacerbated via movement in this population, the encouragement of physical activity among individuals with arthritis should be tailored to their mobility-related personal circumstances and sense of well-being while addressing concerns regarding joint safety [45].

The current study showed that as the number of comorbidities increased, particularly in individuals with ≥1 existing comorbidities, the prevalence of older individuals having long sedentary times significantly increased. This finding is consistent with a previous study that showed that the prevalence of high sedentary behaviour linearly increased in middle-aged and older adults based on the number of chronic conditions [46]. Furthermore, our study indicated that independent positive associations existed between the number of comorbidities and long sedentary times. A cross-sectional study conducted in the United Kingdom reported similar results, suggesting an independent association between physical multimorbidity and sedentary behaviour [24]. A study conducted in the United States showed that for every 60 min/day increase in sedentary behaviour, participants aged ≥20 years had 11% increased odds of multimorbidity (with ≥2 morbidities) [23]. Given the beneficial effects of reducing sedentary behaviour in older adults with chronic conditions, older adults with multimorbidity are a major concern for clinicians. However, based on the current cross-sectional study, it is not possible to determine whether mobility restrictions caused by multimorbidity lead to prolonged sedentary behaviour or multiple diseases occur in older individuals who have sedentary lifestyles. Therefore, prospective studies are needed to identify causal relationships between multimorbidity and sedentary behaviour in the older population.

The principal conclusion that we reached based on our results was that multiple sociodemographic, behavioural, and health factors were independently associated with sedentary behaviour in Korean older individuals that were previously unknown. By employing a behaviour-specific perspective, ecologic models of health behaviour can integrate diverse research findings across six clusters of determinants of sedentary behaviour [8,9]. Considering the lack of research into markers of sedentary behaviour in older adults, the results of the current study can contribute to the future development of a robust ecologic model of sedentary behaviour in the older population. Recent studies have consistently suggested that replacing sedentary behaviour with light or moderate-to-vigorous physical activity leads to better health outcomes, including improved muscle function and cardiometabolic health [47,48]. By considering factors associated with sedentary behaviour and ecologic models for sedentary behaviour, healthcare professionals can identify high-risk groups with prolonged sedentary times in the older population and develop appropriate intervention strategies. Through a balanced approach to physical activity, sedentary behaviour, and sleep, better health outcomes will be achieved for the older population.

Our study had several limitations. Firstly, causal relationships cannot be determined based on the study due to its cross-sectional design. Secondly, sedentary behaviour and physical activity were assessed using questionnaires rather than objective measurements. Although using both subjective and objective measures captures important aspects of sedentary behaviour, subjective self-reported measures of sedentary behaviour are easy, cost-effective, and do not affect habitual behaviour, making such measures important tools in large-scale population-based studies [49]. Thirdly, KNHANES does not assess all chronic health conditions that affect older adults and includes only prevalent diseases. In addition, information regarding the severity of chronic diseases is not recorded in KNHANES. Finally, this study was conducted with Korean participants, making it difficult to generalise the results to other ethnicities and countries.

## 5. Conclusions

A wide range of sociodemographic, behavioural, and health factors are independently associated with long sedentary times in older adults. The established factors associated with sedentary behaviour are crucial for the development of customised interventions to mitigate health risks and encourage active lifestyles in the older population. By understanding these factors, healthcare professionals can help older individuals lead a healthy lifestyle as they age. Future prospective studies are needed to determine causal relationships between sociodemographic, behavioural, and health factors and sedentary behaviour. In addition, through future studies that compare participants with long sedentary times to propensity score-matched controls or apply stratification based on sedentary behaviour status and other major factors, novel insights into the causal inference of risk factors associated with sedentary behaviour may be drawn.

## Figures and Tables

**Table 1:** Characteristics of community-dwelling older adults based on sedentary behaviour status.

Variables	Total Participants	Short Sedentary Time	Long Sedentary Time	*p* Value
Unweighted number (n)	8273	3663	4610	
Weighted number (n)	6,030,990	2,622,031	3,408,959	
**Sociodemographic factors**				
Age groups				<0.001
65–69	34.32 (0.66)	40.71 (0.96)	29.40 (0.84)	
70–74	26.13 (0.55)	27.97 (0.83)	24.72 (0.73)	
75–79	23.67 (0.58)	21.31 (0.86)	25.49 (0.76)	
≥80	15.88 (0.52)	10.01 (0.62)	20.39 (0.75)	
Sex				<0.001
Men	43.72 (0.56)	47.39 (0.88)	40.89 (0.77)	
Women	56.28 (0.56)	52.61 (0.88)	59.11 (0.77)	
Education				0.978
≤9 years	72.14 (0.78)	72.16 (1.02)	72.13 (0.98)	
>9 years	27.86 (0.78)	27.84 (1.02)	27.87 (0.98)	
Occupation				<0.001
Employed	32.83 (0.73)	38.92 (1.08)	28.16 (0.86)	
Unemployed	67.17 (0.73)	61.08 (1.08)	71.84 (0.86)	
Marital status				0.059
Married	99.28 (0.10)	99.49 (0.11)	99.13 (0.15)	
Unmarried	0.72 (0.10)	0.51 (0.11)	0.87 (0.15)	
Household composition				<0.001
Living with other	80.21 (0.58)	83.56 (0.68)	77.62 (0.79)	
Living alone	19.79 (0.58)	16.44 (0.68)	22.38 (0.79)	
Household income				<0.001
Low	45.88 (0.88)	43.23 (1.13)	47.91 (1.09)	
Lower-middle	27.45 (0.69)	29.40 (0.95)	25.94 (0.86)	
Upper-middle	16.03 (0.60)	17.24 (0.83)	15.10 (0.72)	
High	10.65 (0.59)	10.13 (0.68)	11.05 (0.75)	
Residence				0.005
Rural	24.03 (1.57)	26.52 (1.89)	22.11 (1.63)	
Urban	75.97 (1.57)	73.48 (1.89)	77.89 (1.63)	
**Behavioural factors**				
Alcohol consumption				0.956
Non-excessive	93.70 (0.32)	93.72 (0.47)	93.68 (0.42)	
Excessive	6.30 (0.32)	6.28 (0.47)	6.32 (0.42)	
Smoking habits				0.179
Never	61.86 (0.58)	60.60 (0.93)	62.83 (0.77)	
Past	28.52 (0.54)	29.37 (0.86)	27.87 (0.71)	
Current	9.62 (0.39)	10.03 (0.56)	9.30 (0.52)	
Aerobic exercise				<0.001
Sufficient	33.86 (0.69)	42.27 (0.96)	27.44 (0.86)	
Insufficient	66.14 (0.69)	57.73 (0.96)	72.56 (0.86)	
Resistance exercise				0.001
Sufficient	17.79 (0.53)	19.87 (0.88)	16.19 (0.65)	
Insufficient	82.21 (0.53)	80.13 (0.88)	83.81 (0.65)	
**Health factors**				
Weight level				<0.001
Underweight	2.90 (0.22)	2.68 (0.30)	3.08 (0.30)	
Normal weight	33.94 (0.64)	36.44 (0.99)	32.00 (0.80)	
Overweight	26.23 (0.58)	26.48 (0.88)	26.04 (0.77)	
Obese	36.92 (0.64)	34.40 (0.96)	38.88 (0.83)	
Hypertension	62.99 (0.65)	60.45 (0.98)	64.95 (0.86)	<0.001
Diabetes	27.79 (0.58)	25.24 (0.84)	29.76 (0.77)	<0.001
Cardiovascular disease	13.50 (0.43)	11.06 (0.58)	15.37 (0.60)	<0.001
Cancer	10.13 (0.39)	9.28 (0.56)	10.78 (0.54)	0.056
Arthritis	31.78 (0.59)	27.61 (0.84)	34.99 (0.83)	<0.001
Depression	6.22 (0.31)	5.52 (0.44)	6.76 (0.42)	0.039

Values are % (standard errors).

**Table 2:** Sociodemographic factors associated with long sedentary times.

Sociodemographic Factors	Unadjusted OR (95% CI)	Adjusted OR * (95% CI)
Age groups		
65–69	Reference	Reference
70–74	1.22 (1.08–1.39)	1.15 (1.01–1.31)
75–79	1.66 (1.44–1.91)	1.48 (1.26–1.73)
≥80	2.82 (2.38–3.35)	2.49 (2.05–3.01)
Sex		
Men	Reference	Reference
Women	1.30 (1.18–1.43)	1.32 (1.11–1.57)
Education		
≤9 years	Reference	Reference
>9 years	1.00 (0.89–1.13)	1.22 (1.07–1.41)
Occupation		
Employed	Reference	Reference
Unemployed	1.63 (1.45–1.82)	1.23 (1.09–1.38)
Marital status		
Married	Reference	Reference
Unmarried	1.71 (0.97–3.01)	1.76 (0.95–3.28)
Household composition		
Living with other(s)	Reference	Reference
Living alone	1.47 (1.30–1.65)	1.24 (1.08–1.43)
Household income		
Low	Reference	Reference
Middle-low	0.80 (0.70–0.90)	1.00 (0.88–1.15)
Middle-high	0.79 (0.68–0.92)	0.96 (0.81–1.13)
High	0.98 (0.82–1.18)	1.24 (1.01–1.51)
Residence		
Rural	Reference	Reference
Urban	1.27 (1.08–1.50)	1.35 (1.14–1.61)

Values are presented as odds ratios (OR) (95% confidence intervals [CI]). * Adjusted for alcohol, smoking, aerobic exercise, resistance exercise, obesity, hypertension, diabetes, cardiovascular disease, cancer, arthritis, depression, and all variables in the first column.

**Table 3:** Behavioural and health factors associated with long sedentary times.

Variables	Unadjusted OR (95% CI)	Adjusted OR * (95% CI)
**Behavioural factors**		
Alcohol consumption		
Non-excessive	Reference	Reference
Excessive	1.01 (0.82–1.24)	0.81 (0.65–1.02)
Smoking habits		
Never	Reference	Reference
Past	0.92 (0.82–1.02)	1.23 (1.03–1.47)
Current	0.89 (0.76–1.06)	1.26 (1.02–1.57)
Aerobic exercise		
Sufficient	Reference	Reference
Insufficient	1.94 (1.74–2.16)	1.80 (1.60–2.02)
Resistance exercise		
Sufficient	Reference	Reference
Insufficient	1.28 (1.11–1.48)	1.01 (0.86–1.18)
**Health factors**		
Weight level		
Underweight	1.31 (0.98–1.75)	1.12 (0.83–1.52)
Normal weight	Reference	Reference
Overweight	1.12 (0.98–1.28)	1.17 (1.01–1.34)
Obese	1.29 (1.14–1.46)	1.27 (1.12–1.45)
Hypertension	1.21 (1.09–1.35)	1.01 (0.90–1.14)
Diabetes	1.26 (1.12–1.40)	1.17 (1.04–1.32)
Cardiovascular disease	1.46 (1.26–1.69)	1.30 (1.11–1.52)
Cancer	1.18 (0.99–1.40)	1.19 (0.99–1.43)
Arthritis	1.41 (1.26–1.57)	1.26 (1.11–1.43)
Depression	1.24 (1.01–1.53)	1.16 (0.94–1.44)

Values are presented as odds ratios (OR) (95% confidence intervals [CI]). * Adjusted for age, sex, education, occupation, marital status, household composition, household income, residence, and all variables in the first column.

## Data Availability

Data are available to the public on the KNHANES website (https://knhanes.kdca.go.kr/knhanes, accessed on 10 January 2023).
